# Identification of Differentially Expressed miRNAs between White and Black Hair Follicles by RNA-Sequencing in the Goat (*Capra hircus*)

**DOI:** 10.3390/ijms15069531

**Published:** 2014-05-28

**Authors:** Zhenyang Wu, Yuhua Fu, Jianhua Cao, Mei Yu, Xiaohui Tang, Shuhong Zhao

**Affiliations:** 1 Key Laboratory of Agricultural Animal Genetics, Breeding and Reproduction, Ministry of Education, Huazhong Agricultural University, Wuhan 430070, Hubei, China; E-Mails: wuzhenyang0724@gmail.com (Z.W.); yhfu2012@gmail.com (Y.F.); jhcao@mail.hzau.edu.cn (J.C.); yumei@mail.hzau.edu.cn (M.Y.); 2 Agriculture and Animal Husbandry College of Tibet University, Linzhi 860000, Tibet, China; E-Mail: xiaoyuhong515@hotmail.com

**Keywords:** goat, coat color, RNA-sequencing, hair follicle, miRNA

## Abstract

MicroRNAs (miRNAs) play a key role in many biological processes by regulating gene expression at the post-transcriptional level. A number of miRNAs have been identified from livestock species. However, compared with other animals, such as pigs and cows, the number of miRNAs identified in goats is quite low, particularly in hair follicles. In this study, to investigate the functional roles of miRNAs in goat hair follicles of goats with different coat colors, we sequenced miRNAs from two hair follicles samples (white and black) using Solexa sequencing. A total of 35,604,016 reads were obtained, which included 30,878,637 clean reads (86.73%). MiRDeep2 software identified 214 miRNAs. Among them, 205 were conserved among species and nine were novel miRNAs. Furthermore, DESeq software identified six differentially expressed miRNAs. Quantitative PCR confirmed differential expression of two miRNAs, miR-10b and miR-211. KEGG pathways were analyzed using the DAVID website for the predicted target genes of the differentially expressed miRNAs. Several signaling pathways including Notch and MAPK pathways may affect the process of coat color formation. Our study showed that the identified miRNAs might play an essential role in black and white follicle formation in goats.

## 1. Introduction

Coat color has long been a subject of interest to breeders and scientists [1]. Not only is coat color a model phenotype for studying gene action and gene interactions, but also is important for goatskin, which is a valuable animal product. Mammalian coat color is almost totally dependent on either the presence or absence of melanin in skin and follicles [2]. Therefore, it is necessary to understand the process of melanocyte formation. Melanocytes emerge from the neural crest, which is an early embryonic structure [3]. In the late embryonic stage, neural crest cells differentiate into melanoblasts, which migrate to the skin basal layer, where they settle and are involved in the development of the hair follicles [4]. The pigment cells reside in the bulb of the hair follicle and affect the coat color. Various factors affect mammalian coat color, including the composition, numbers, and arrangements of the melanin granules [5]. Moreover, a number of genes such as *TYR*, *MITF*, *ASIP* and *MC1R* regulate the progress of hair follicle pigmentation. However, few studies have studied the regulatory mechanisms at the post-transcriptional level. MicroRNAs (miRNAs) are small non-protein-coding transcripts that regulate gene expression post-transcriptionally by binding to the 3'-untranslated region (3'-UTR) of the target messenger RNAs (mRNAs) thereby causing suppression of protein synthesis or mRNA cleavage [6]. Increasing evidence shows that miRNAs play an important regulatory role in a variety of biological processes. The development of next-generation massively sequencing (NGMS) technologies, providing high throughput with low cost, have revolutionized genomic research, allowing many animal miRNAs to be identified and deposited in MiRBase (http://www.mirbase.org/). To date, 24,521 entries representing hairpin precursor miRNAs, expressing 30,424 mature miRNAs products in 206 species have been identified and deposited in the public miRNA database miRBase (Release 20.0, June 2013). Among them, 2578 miRNAs were from human, 1908 from mouse, and 153 from sheep. Only a few studies identified miRNAs in goats (*Capra hircus*) [7,8,9], indicating that goat miRNAs still need to be sequenced.

In the last two years, significant progress has been made on the goat genome. The 2.66 Gb genome sequence data were obtained by combing short-read sequencing data and optical mapping data from a female Yunnan black goat. Meanwhile, 51 differentially expressed genes between the two types of hair follicles, the primary and secondary follicle, of a cashmere goat, were identified by comparative transcriptome analysis [10]. Conserved miRNAs (346) were identified between dry period and peak lactation mammary gland tissues in the dairy goat [11]. Five differentially expressed miRNAs were verified by quantitative PCR in the ovaries of pregnant and non-pregnant goats [12]. Hair color is an important trait in the goat. Recently, several studies have tried to identify genes and miRNAs in goatskin and hair follicles. MiRNA data produced by Solexa sequencing among three follicular cycling stages in goatskin and hair follicles were reported [13]. Similar studies focused on the identification of miRNAs in hair follicle and skin development [14,15,16]. These studies enriched the goat hair follicle and skin miRNA database and enhanced our understanding of the process of miRNA regulation on development of skin and hair follicle. However, very few references are related to the mechanism of how miRNAs regulate coat color. Five differentially expressed miRNAs between the white and brown skin of alpaca were identified by quantitative PCR, including miR-211 and miR-202, which were significantly expressed in brown and white skins, respectively [17]. MiR-137, which can downregulate the microphthalmia-associated transcription factor (MITF) was verified to influence the phenotype of coat color in transgenic mice overexpressing miR-137 [18]. No studies have been attempted to identify miRNAs affecting coat color in the goat.

In this study, we sequenced miRNAs from black and white hair follicles collected from 1-year old crossbreed white and black coat color goats. These results provide new information on miRNA expression profiles in the goat and identify possible miRNA regulated pathways related to pigmentation in hair follicles.

## 2. Results

### 2.1. Overview of Sequencing Data

To identify differentially expressed miRNAs in the two types of hair follicles, two small RNA libraries were constructed for Solexa sequencing. A total of 35,604,016 raw reads and 30,878,637 clean reads were obtained after eliminating the low quality reads and adaptor sequences. The size distribution of the reads was similar between the two libraries. Most of the reads were from 21 to 24 nt. Reads whose sizes were 22 nt accounted for 20.33% and 20.16% of the total sequence reads whereas the reads that were 24 nt accounted for 22.01% and 19.70% in white follicle and black follicle libraries, respectively; the size distribution of the small RNAs from white and black hair follicles was similar.

### 2.2. Identification of Conserved and Novel MiRNAs

**Figure 1:**
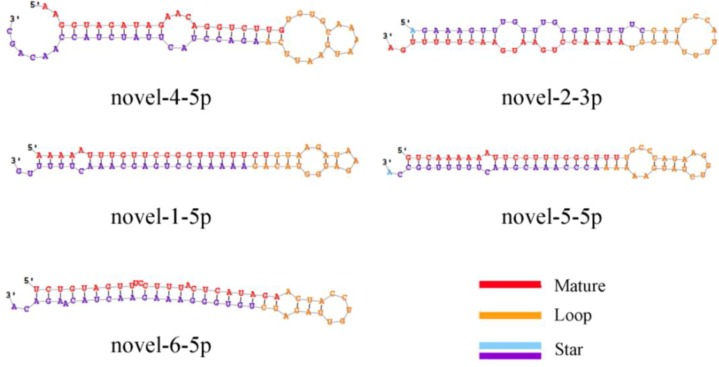
Predicted secondary structures of novel miRNAs. The red color indicates the mature sequence, the yellow color indicates the loop sequence, the blue color indicates the predicted star sequence, and the purple indicates the miRNA star sequences.

To identify conserved miRNAs in goat hair follicles, the reads were compared to the precursors and mature miRNAs in miRBase 20.0 (http://www.mirbase.org). In total, 205 and nine conserved and novel miRNAs were identified, respectively. Among these conserved miRNAs, 168 miRNAs were also identified in cattle and only 37 miRNAs in sheep. All the information is shown in Table S1, The 10 most abundant miRNAs are also listed in Table 1. The precursor sequences and secondary structures of the nine novel miRNAs identified from our sequencing data using miRDeep2 software (Table S2) were predicted. The five most abundantly expressed novel miRNAs are shown in Table 2 , Table 3, and Figure 1. The expression levels of the novel miRNAs were relatively low in our results. A total of 193 conserved and novel miRNAs were co-expressed, eight miRNAs were white follicle-specific and 13 were black follicle-specific.

**Table 1:** The most abundantly expressed miRNAs in goat hair follicles.

MiRNAs name	Normalized expression level		Mature sequences
	WF	BF	
Goat-miR-146b-5p	186,997.77	158,761.10	ugagaacugaauuccauaggcugu
Goat-miR-27b-3p	79,872.78	72,800.46	uucacaguggcuaaguucugc
Goat-miR-205-5p	20,575.80	19,911.95	uccuucauuccaccggagucug
Goat-miR-181a-2-5p	21,177.16	16,613.29	aacauucaacgcugucggugagu
Goat-miR-181a-1-5p	21,176.79	16,613.08	aacauucaacgcugucggugagu
Goat-miR-92a-3p	19,003.38	17,003.44	uauugcacuugucccggccugu
Goat-miR-182-5p	14,218.79	13,630.30	uuuggcaaugguagaacucacacu
Goat-miR-26a-1-5p	14,855.58	12,171.42	uucaaguaauccaggauaggcu
Goat-miR-26a-2-5p	14,837.64	12,152.12	uucaaguaauccaggauaggcu
Goat-let-7f-5p	10,685.28	8870.12	ugagguaguagauuguauaguu

**Table 2:** The five most abundantly expressed novel miRNAs in goat hair follicels.

MiRNAs name	Normalized expression level		Mature sequences
	WF	BF	
Novel-84-3p	195.35	203.44	uccccugcaucuccacca
Novel-9-3p	94.46	84.20	aaaaccugaaugaacuuuugag
Novel-21-3p	58.89	53.72	aaagccugaaugaacuuuuugg
Novel-29-5p	105.15	0	aagguagauagaacaggucuug
Novel-76-5p	42.67	40.61	uauugcacauuacuaaguugc

**Table 3:** Predicted precursor sequences and genome locations of five novel miRNAs.

MiRNAs name	MiRNA precursor sequence	Chromosome	Strand
Novel-84-3p	ggcuacagucugugggguugcagaguuggacacaacugagcacauccccugcaucuccacca	chr8	−
Novel-9-3p	aaaauguucaugcagguuuuuccguaagauguuacaggaaaaccugaaugaacuuuugag	chrX	+
Novel-21-3p	caauaaguucguuuggguuuuuggcuguuacagaaagccugaaugaacuuuuugg	chrX	+
Novel-29-5p	aagguagauagaacaggucuugugugcaaaaugaauucaagaccuacuuaucuaccaacagc	chr21	+
Novel-76-5p	uauugcacauuacuaaguugcauguugucacggccucagugcaauuuagugugugugauauu	chr8	−

WF: white follicles and BF: black follicle.

### 2.3. Identification of Differentially Expressed MiRNAs and Prediction of MiRNA Target Genes, Pathways, and GO Analysis

**Figure 2:**
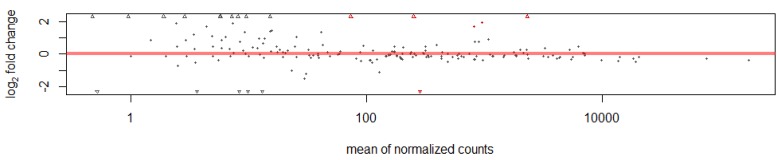
Plot of normalized mean *versus* log2-fold change for the miRNAs in black and white hair follicles. The red colour marks genes detected as differentially expressed. The red points and triangle below and above the red line indicate downregulation and upregulation, respectively in black hair follicles, and the reverse in white hair follicles. The symbols at the upper and lower plot border (the red and dark triangle) indicate genes with very large or infinite log fold change.

The DESeq package analyzed the differentially expressed miRNAs and identified six miRNAs. Five miRNAs in the white hair follicle were significantly upregulated, while one miRNA significantly downregulated (Figure 2). Normalized expression levels in the white and black hair follicles of the differentially expressed miRNAs are listed in Table 4.

**Table 4:** Differentially expressed miRNAs among different goat follicle tissues.

MiRNAs	Normalized expression level		*p* value
	WF	BF	
Goat-miR-10b-5p	0	64,534.56	2.36 × 10^−121^
Goat-miR-1307-5p	7796.83	0	6.98 × 10^−25^
Goat-miR-146a-5p	0	2054.46	2.77 × 10^−7^
Goat-miR-143-3p	5469.10	20,954.76	1.14 × 10^−5^
Goat-miR-30a-5p	5363.49	17,292.17	2.69643 ×10^−4^
Goat-miR-211-5p	0	7016.16	3.84 × 10^−22^

**Figure 3:**
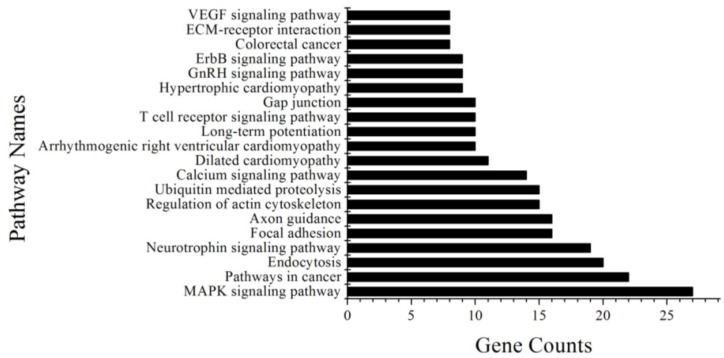
Top 20 pathways predicted to be targeted by differentially expressed miRNAs.

To understand the function of the differentially expressed miRNAs in goat hair follicles, we predicted the target genes of the miRNAs using The Target Scan website, PicTar and DIANA-microT v3.0, and counted miRNAs that were predicted by two of these software tools. As a result, 981 genes were found to be targeted by five differentially expressed miRNAs, except miR-1307 (Table S3). These genes were analyzed by the DAVID website, identifying 26 pathways that could be involved in regulation of coat color (Table S4). We listed the 20 pathways for which the gene count was more than 1% in Figure 3. Among these pathways, the mitogen-activated protein kinase (MAPK) signaling pathway regulates cell proliferation and differentiation, and can induce MITF, which increases the production of tyrosinase [19]. The MAPK signaling pathway is involved in 27 of the genes, which are targeted by the five miRNAs (Table 5).

**Table 5:** Target gene of differentially expressed miRNAs in the MAPK signaling pathway.

MiRNA name	Target gene in MAPK signaling pathway
MiR-10b	*BDNF*
MiR-211	*SOS1*
MiR-143	*CACNA1E*, *FGF1*, *MAPK7*, *MAP3K7*, *PDGFRA*, *KRAS*
MiR-30a	*RAP1B*, *RASA1*, *RAPGEF2*, *TAOK1*, *CACNB2*, *CASP3*, *CACNA1C*, *IL1A*, *MAP2K4*, *MAP3K1*, *MAP3K12*, *MAP3K2*, *MAP3K5*, *NF1*, *PPP3CA*, *PPP3CB*, *RPS6KA2*, *CRKL*
MiR-146a	*TRAF6*

To gain an insight into the molecular functions of genes in biological processes, we annotated the genes targeted by differentially expressed miRNAs using GO categories (Table S5). The five most enriched GO categories contained at least 10% of the total predicted target genes in the biological processes analysis. Most genes were enriched in two types of functions for molecular function: binding activity and transcription activity. The 10 most enriched GO categories are listed in Table 6, Table 7 and Table 8.

Expression of tyrosinase gene (TYR) and related genes, such as tyrosinase-related proteins-1 (*TYRP1*), is the hallmark of mammalian melanocytes [20]. The biosynthesis of the pigment itself is a process orchestrated by the concerted action of tyrosinase, tyrosinase-related protein-1 and dopachrome tautomerase. They are melanocyte-specific enzymes that act to synthesize two types of pigment, eumelanin (black/brown) and/or pheomelanin (yellow/red), which are then deposited in discrete membrane-bound organelles, known as melanosomes [21,22]. The proportion of the two types of pigment result in different coat colors, such as red, yellow, brown or white. If the process of pigment synthesize are blocked directly or indirectly, the coat color will change.

**Figure 4:**
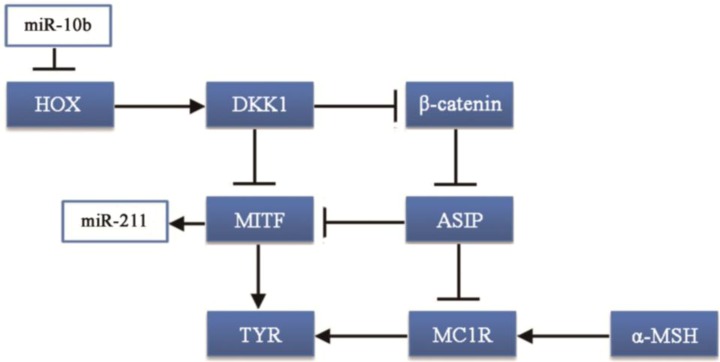
The possible regulatory pathway of melanogesis activation.

The possible regulatory pathways of the differentially expressed miRNAs were built according to the results from our study and a literature search (Figure 4).

**Table 6:** The 10 most enriched GO categories in biological process.

GO accession	GO terms	Gene count	Percentage (%)	*p* value
GO:0006357	regulation of transcription	188	23.76738306	1.04 × 10^−^^10^
GO:0045449	transcription	151	19.0897598	2.51 × 10^−^^8^
GO:0010557	regulation of RNA metabolic process	128	16.18204804	1.18 × 10^−^^6^
GO:0045893	regulation of transcription, DNA-dependent	125	15.80278129	1.77 × 10^−^^6^
GO:0051254	intracellular signaling cascade	86	10.87231353	2.94 × 10^−^^4^
GO:0031328	regulation of transcription from RNA polymerase II promoter	79	9.987357775	3.36 × 10^−^^12^
GO:0010629	positive regulation of macromolecule metabolic process	78	9.860935525	1.89 × 10^−^^8^
GO:0009891	regulation of apoptosis	69	8.723135272	1.28 × 10^−^^6^
GO:0045941	regulation of programmed cell death	69	8.723135272	1.81 × 10^−^^6^
GO:0010628	regulation of cell death	69	8.723135272	2.06 × 10^−^^6^

**Table 7:** The 10 most enriched GO categories in cellular component.

GO accession	GO terms	Gene count	Percentage (%)	*p* value
GO:0043232	intracellular non-membrane-bounded organelle	132	16.687737	0.00202852
GO:0043228	non-membrane-bounded organelle	132	16.687737	0.00202852
GO:0044459	plasma membrane part	110	13.9064475	0.009611689
GO:0031974	membrane-enclosed lumen	99	12.5158028	0.002133467
GO:0043233	organelle lumen	97	12.2629583	0.00251202
GO:0070013	intracellular organelle lumen	96	12.136536	0.001867367
GO:0031981	nuclear lumen	90	11.3780025	2.40 × 10^−^^5^
GO:0000267	cell fraction	78	9.86093552	4.47 × 10^−^^7^
GO:0005626	insoluble fraction	70	8.84955752	7.38 × 10^−^^9^
GO:0005624	membrane fraction	69	8.72313527	3.94 × 10^−^^9^

**Table 8:** The 10 most enriched GO categories in molecular function.

GO accession	GO terms	Gene count	Percentage (%)	*p* value
GO:0043167	ion binding	242	31	8.45 × 10^−^^4^
GO:0046872	metal ion binding	240	30	3.30 × 10^−^^4^
GO:0043169	cation binding	240	30	6.09 × 10^−^^4^
GO:0046914	transition metal ion binding	167	21	0.001082879
GO:0003677	DNA binding	156	20	7.00 × 10^−^^6^
GO:0008270	zinc ion binding	146	18	2.68 × 10^−^^4^
GO:0000166	nucleotide binding	132	17	0.008790377
GO:0030528	transcription regulator activity	126	16	4.59 × 10^−^^10^
GO:0003700	transcription factor activity	85	11	9.09 × 10^−^^8^
GO:0043565	sequence-specific DNA binding	50	6.3	2.57 × 10^−^^4^

### 2.4. Validation of the Sequencing Data by Quantitative PCR (qPCR)

**Figure 5:**
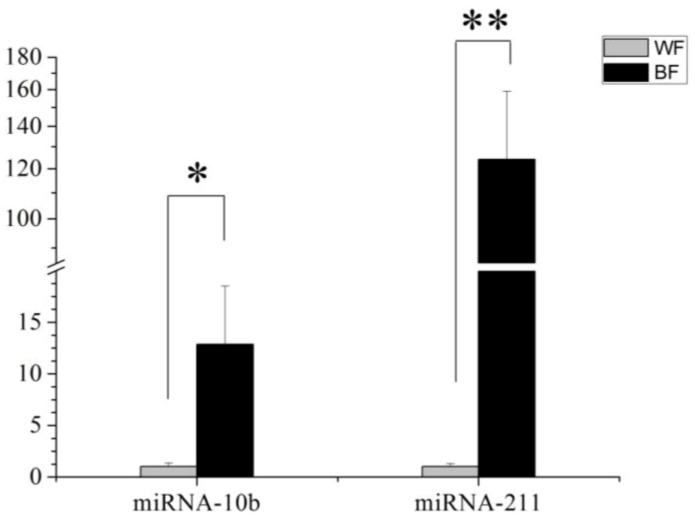
The expression of miR-211 and miR-10b in two tissues. MiR-211 is highly expressed in black hair follicle (BF) compared with the white hair follicle (WF). Results are the mean ± SD from triplicate determinations. *p* < 0.01(**). MiR-10b is highly expressed in black hair follicles (BF) in compared with the white hair follicles (WF). Results are the mean ± SD from triplicate determinations. *p* < 0.05(*).

To verify the sequencing results, the differentially expressed miRNAs were detected by qPCR analyses. The expressions of two miRNAs, miR-10b and miR-211, were confirmed (Figure 5). The result is consistent with the sequencing data. The expression levels of miR-10b and miR-211 were significantly higher in black follicles than in white follicles.

## 3. Discussion

This study identified 205 conserved miRNAs and nine novel miRNAs by RNA-Seq in goat hair follicles. Six differentially expressed miRNAs were predicted in two types of hair follicles tissues. Most miRNAs were upregulated in black follicles, only miR-1307 was downregulated in white hair follicle. However, the expression of the novel miRNA was too low to perform further detection. Two differentially expressed miRNAs, miR-10b and miR-211, were verified by qPCR. Our results thus offer new information on goat hair follicle expressed miRNAs.

We obtained 214 miRNAs in this study. However, only a few miRNAs were differentially expressed between white and black hair follicles. One reason may be that the samples we used were from adult goats. Most of the relevant biological processes such as melanoblast differentiation, migration and maturation are completed during embryonic development. Thus, more differentially expressed miRNAs might be identified during early development than in the adult hair follicles where miRNAs may be involved in fewer biological events. In adult hair follicles, genes and miRNAs are probably involved in more functions related to melanocyte stem cell differentiation into melanocytes or the proliferation of melanocytes. Nevertheless, the identification of miRNAs in skin add a new dimension in the regulatory networks and identified novel players in hair follicle color formation [23]. Our results may prompt further studies on how miRNAs affect hair follicle development, differentiation and pigmentation.

In our study, the MAPK signaling pathway was the major pathway involving 27 genes, and targeted by five differentially expressed miRNAs including miR10b. The MAPK family proteins, such as p38, ERK and JNK, play critical roles in melanogenesis [24]. Most studies reported that the p38 MAPK signaling pathway activates MITF, which can up-regulate the expression of melanogenic enzymes [25]. However, the ERK and/or JNK/SAPK pathways cause down-regulation of melanin synthesis by downregulating MITF [26]. The detailed mechanism involving p38 MAPK in melanin synthesis is not completely understood.

MiR-10b was one of the most abundant and differentially expressed miRNA in black hair follicles, with approximately 64,534.56 reads. MiR-10b takes part in carcinogenesis: miR-10b can suppress the translation of the HOXD10 gene leading to increased RHOC expression and AKT phosphorylation [27,28]. Although currently there are no studies on the impact of the HOXD10 gene on melanocytes or the production of melanin, many studies show that genes in the HOX gene family are related to the development of hair follicles, especially HOXC13 [29,30]. Thus, the HOX gene family may be associated with coat color formation. Moreover, the HOXA10 gene can upregulate the Dickkopf 1 (DKK1) gene [31], which regulates skin pigmentation. DKK1 can inhibit the function and proliferation of melanocytes by suppressing β-catenin and microphthalmia-associated transcription factor (MITF) [32,33], which can promote the synthesis of melanin.

Our results showed that miR-10b regulates the *DVL3* gene in the Notch pathway. Notch is an evolutionarily conserved local cell-signaling pathway that participates in a variety of cellular processes such as cell fate specification, differentiation, proliferation, apoptosis, adhesion, epithelial-mesenchymal transition, migration and angiogenesis [34], and the development of hair follicles [35]. Melanocytes produce melanin and are tightly linked with hair regeneration cycles [36]. In the hair follicle, melanocyte and melanocytes stem cells numbers are maintained in a dynamic balance. In the cell cycle of the hair follicle, melanocytes proliferate during the hair growth phase and are depleted during the regression phase; the new melanocyte is produced by the differentiation and proliferation of melanocyte stem cells [37,38,39]. The Notch signaling pathway plays a key role in melanoblasts, melanocyte stem cells, keratinocytes and melanocytes [40]. Many studies reported that lack of Notch signaling can lead to the reduction of the number of melanocytes which can cause the coat color [41,42,43]. Interestingly, HOX, the Notch signaling pathway and the Wnt/β-catenin signaling pathway interact via cross-talk [44,45]. Taken together, miR-10b could be an important regulator in goat coat color formation.

MiR-211 had a similar expression pattern to miR-10b. Most studies of this miRNA are on cancer, with few reports on hair follicle development or coat color. One study reported that miR-211 is highly expressed in brown alpaca skin via white alpaca skin expression [17]. That result is consistent with our study, however, the mechanism has not been investigated. MITF promotes the expression of many genes in pigment cell and regulates melanocyte development by increasing the expression of enzymes, involved in melanin synthesis and melanosome biogenesis [46]. Studies have shown that miR-211 is induced by the expression of MITF [47,48]. This could explain why the expression of miR-211 is higher in black follicles than in white follicles. However, how miR-211 regulates melanocytes or melanin synthesis is not clear.

## 4. Experimental Section

### 4.1. Sample Collection, RNA Extraction and Library Construction

**Figure 6:**
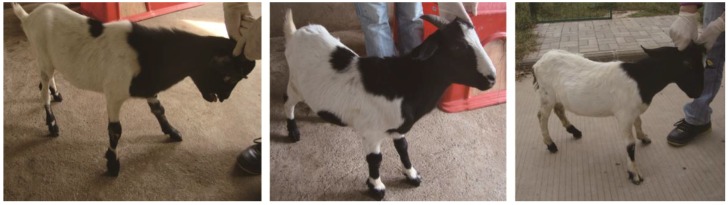
The white and black hair follicles were collected from three 1-year old crossbred black and white goats.

To eliminate the genetic background, samples were collected from three 1-year old crossbreed white and black coat colored goats (Figure 6). Black hair and white hair were pulled out with their follicles, the hair shaft was removed with scissors and transferred to 1.5 mL RNase-free tube with 1 mL Trizol reagent (Life Technologies Corporation, Carlsbad, CA, USA). The samples were homogenized and then stored at −80 °C.

Total RNA was isolated from hair follicles of goats according to the manufacturer’s protocol. Quality and quantity of RNA was examined using a NanoDrop 2000/2000C (Thermo Fisher Scientific Inc., Waltham, MA, USA) and integrity was detected using agarose gel electrophoresis. Two samples (one from white hair follicle tissue and one from black hair follicle tissue) from one goat each were sent to Genergy Biotechnology Co., Ltd. (Shanghai, China) for small RNA library construction and sequencing. Sequencing was performed using an Illumina HiSeq 2000 Genome Analyzer (Illumina Inc., Santiago, CA, USA). Libraries were constructed using a TruSeq Small RNA Sample Preparation kit (Illumina Inc., Santiago, CA, USA).

### 4.2. Identification of Conserved and Novel MiRNAs

The quality of the original raw sequencing data obtained by Solexa deep sequencing was assessed using FastQC software (http://www.bioinformatics.babraha m.ac.uk/projects/fastqc/). The clean reads were obtained by trimming the low-quality reads and eliminating reads with contaminants, e.g., reads without 3' primer and reads shorter than 18 nt. MiRDeep2 software [49,50] was used to predict the known and novel miRNAs. The parameter used to screen for “novel” miRNAs predicted using miRDeep2 were as follows: (a) Delete miRDeep2 score: <100; (b) The ratio of mature miRNA *vs.* miRNA*; and (c) we screened the predicted miRNAs strictly according to the hairpin structure, with only a 2-nt overhang, which is the hallmark of a bona fide miRNA.

The expression level of each miRNA was normalized by the following formula: Normalized expression (NE) = Actual miRNA count/Total count of clean reads × 1,000,000. We removed the miRNAs with a normalized expression level lower than 1 and an estimated probability value lower than 0.95.

### 4.3. Identification and Validation of Differentially Expressed MiRNAs

The package DESeq [51] was used to identify differentially expressed miRNAs. This software provides methods to detect miRNA differential expression using the negative binomial distribution and a shrinkage estimator for the distribution’s variance.

Differentially expressed miRNAs were confirmed with qPCR [52]. Six samples (three white follicle samples and three black follicle samples) from three goats were used in qPCR analysis. The miRNA specific primers are shown in Table 9. One microgram of total RNA from each sample was reverse-transcribed into cDNA using the Thermo Scientific Revert Aid First Strand cDNA Synthesis Kit (Thermo Fisher Scientific Inc., Waltham, MA, USA). U6 snRNA was used as the internal control. QPCR was performed using standard protocols on the Roche LightCycler 480 Real-Time PCR Detection System (Hoffmann-La Roche Ltd, Basel, Switzerland). The 2^−ΔΔ*C*t^ method was used to analyze the expression levels [53].

**Table 9:** MiRNA and gene primers.

MiRNA	Primer sequences
Goat-miR-211	Forward: TCGGCAGGTCCCTTTGTCATCC
	Reverse: TGCAGGTCAACTGGTGTCGT
	Loop prime: CTCAACTGGTGTCGTGGAGTCGGCAATTCAGTTGAGTGGGCAAA
Goat-miR-10b	Forward: TCGGCAGGACCCTGTAGAACCG
	Reverse: TGCAGGTCAACTGGTGTCGT
	Loop prime: CTCAACTGGTGTCGTGGAGTCGGCAATTCAGTTGAGCACAAATT
U6	Forward: CTCGCTTCGGCAGCACA
	Reverse: AACGCTTCACGAATTTGCGT

### 4.4. Prediction of MiRNA Target Genes, Pathways and GO Analysis

The TargetScan website (http://www.targetscan.org/), PicTar (http://pictar.mdc-berlin.de/) and DIANA-microT v3.0 (http://diana.cslab.ece.ntua.gr/microT/) were used to predict the targets of differentially expressed miRNAs The DAVID website [54,55] was used to analyze the KEGG pathways, with the following parameters: Count = 2 and EASE = 0.1. GO categories were also analyzed using the DAVID website with the following parameters: Count = 10 and EASE = 0.01.

## 5. Conclusions

In conclusion, the development of hair follicles and melanocytes is a complex event involving numerous genes and pathways that interact and show cross-talk. We showed evidence that miRNAs could also be involved in these processes. Understanding the regulatory mechanism at the post-transcription level will provide new insights into the regulation of coat color formation.

## Supplementary Files

Supplementary File 1 Supplementary Information (XLSX, 203 KB)
